# Transcriptional Dynamics of DNA Damage Responsive Genes in Circulating Leukocytes during Radiotherapy

**DOI:** 10.3390/cancers14112649

**Published:** 2022-05-26

**Authors:** Lourdes Cruz-Garcia, Farah Nasser, Grainne O’Brien, Jakub Grepl, Volodymyr Vinnikov, Viktor Starenkiy, Sergiy Artiukh, Svetlana Gramatiuk, Christophe Badie

**Affiliations:** 1 Centre for Radiation, Chemical and Environmental Hazards, UK Health Security Agency, Chilton, Didcot OX11 0RQ, UK; fagife@gmail.com (F.N.); grainne.obrien@phe.gov.uk (G.O.); Christophe.badie@phe.gov.uk (C.B.); 2 Department of Oncology and Radiotherapy, University Hospital, 500 05 Hradec Kralove, Czech Republic; jakub.grepl@fnhk.cz; 3 Department of Radiobiology, Faculty of Military Health Sciences, Hradec Kralove, University of Defence, 662 10 Brno, Czech Republic; 4 Grigoriev Institute for Medical Radiology and Oncology of the National Academy of Medical Science of Ukraine, 61024 Kharkiv, Ukraine; vlad.vinnikov@ukr.net (V.V.); starenkiy.victor@gmail.com (V.S.); artiukhsergii@ukr.net (S.A.); 5 Ukraine Association of Biobank, 61000 Kharkiv, Ukraine; gramatyuk@ukr.net; 6 Environmental Research Group within the School of Public Health, Faculty of Medicine at Imperial College of Science, Technology and Medicine, London SW7 2BX, UK

**Keywords:** *FDXR*, radiation exposure, biomarkers, PBMCs, radiotherapy, ionizing radiation

## Abstract

**Simple Summary:**

In this study, the transcriptional response of a panel of radiation responsive genes was monitored over time in blood samples after radiation exposure in vivo. For this aim, cancer patients treated by radiotherapy were recruited after consent forms were obtained. Following the first fraction of radiotherapy, 2 mL blood samples were collected at different time points during the first 24h hours (before the second fraction was delivered) and at mid and end of treatment. Amongst the 9 genes studied, the gene *FDXR* stood out as the most sensitive and responsive to the low dose of radiation received from the localised radiation treatment by the circulating white blood cells. The activation of *FDXR* was found to depend on the volume of the body exposed with a peak of expression around 8–9 hours after irradiation was delivered. Finally results obtained ex vivo confirmed the results obtained in vivo.

**Abstract:**

External beam radiation therapy leads to cellular activation of the DNA damage response (DDR). DNA double-strand breaks (DSBs) activate the ATM/CHEK2/p53 pathway, inducing the transcription of stress genes. The dynamic nature of this transcriptional response has not been directly observed in vivo in humans. In this study we monitored the messenger RNA transcript abundances of nine DNA damage-responsive genes (*CDKN1A*, *GADD45*, *CCNG1*, *FDXR*, *DDB2*, *MDM2*, *PHPT1*, *SESN1*, and *PUMA*), eight of them regulated by p53 in circulating blood leukocytes at different time points (2, 6–8, 16–18, and 24 h) in cancer patients (lung, neck, brain, and pelvis) undergoing radiotherapy. We discovered that, although the calculated mean physical dose to the blood was very low (0.038–0.169 Gy), an upregulation of Ferredoxin reductase (*FDXR*) gene transcription was detectable 2 h after exposure and was dose dependent from the lowest irradiated percentage of the body (3.5% whole brain) to the highest, (up to 19.4%, pelvic zone) reaching a peak at 6–8 h. The radiation response of the other genes was not strong enough after such low doses to provide meaningful information. Following multiple fractions, the expression level increased further and was still significantly up-regulated by the end of the treatment. Moreover, we compared *FDXR* transcriptional responses to ionizing radiation (IR) in vivo with healthy donors’ blood cells exposed ex vivo and found a good correlation in the kinetics of expression from the 8-hours time-point onward, suggesting that a molecular transcriptional regulation mechanism yet to be identified is involved. To conclude, we provided the first in vivo human report of IR-induced gene transcription temporal response of a panel of p53-dependant genes. FDXR was demonstrated to be the most responsive gene, able to reliably inform on the low doses following partial body irradiation of the patients, and providing an expression pattern corresponding to the % of body exposed. An extended study would provide individual biological dosimetry information and may reveal inter-individual variability to predict radiotherapy-associated adverse health outcomes.

## 1. Introduction

Radiotherapy (RT) represents a cost-effective tool in cancer treatment alongside surgery and chemotherapy, with roughly half of patients undergoing RT as part of their treatment; this represents 134,000 radiotherapy “episodes” delivered every year for England alone [1]. With more than 14 million new cases of cancer diagnosed globally each year and numbers continuing to rise, RT will continue to be an essential element of treatment [2]. While the tumour is targeted by the radiation beam and receives the highest dose, surrounding normal tissue and circulating white blood cells receive lower doses, potentially leading to long term adverse effects such as cancer [3]. Individual responses of healthy tissues are still not well understood and can lead to dramatic outcomes; for Ataxia Telangiesctasia (AT) patients [4], X-ray exposure should be limited for diagnostic purposes and used only in rare circumstances and at reduced doses for therapy [5,6]. Non-AT patients without clear clinical symptoms before the beginning of RT can also experience severe acute normal tissue toxicity reactions such as those associated with major DNA double-strand break repair deficiency [7,8,9,10,11]. The role of Ataxia-telangiectasia mutated (ATM) seems crucial [12], but biomarkers to identify patients at risk are still needed to allow for more individualised RT treatments [13]. There is still a lack of fully validated biomarkers to assess the acute and late toxicity in addition to long-term risks on an individual basis [14,15,16], particularly in the case of paediatric RT. RT can induce lymphopenia (loss of lymphocytes, the white blood cells associated with immune response) with poor survival after radiotherapy [17]. Moreover, although radiotherapy is mostly a local therapy, it has systemic effects mainly influencing immune and inflammation processes with consequences in the long-term prognosis [18,19].

Radiation-induced cellular damage rapidly activates multiple signal pathways; amongst them the DDR which contribute to cell survival and therapeutic outcome at cellular and patient levels, respectively. The cellular response is coordinated by kinase-signaling cascades; amongst them, the ATM/CHEK2/p53 is activated by induced DNA DSBs [20] leading to p53 stabilisation and activation of its transcription factor functions [21]. P53 induces the transcription of multiple genes [22,23] including *FDXR*, Cyclin G1 (*CCNG1*), cyclin-dependent kinase inhibitor 1A (*CDKN1*), Sestrin 1 (*SESN1*), p53-upregulated modulator of apoptosis (*PUMA*), damage-specific DNA-binding protein 2 (*DDB2*), *MDM2* proto-oncogene (*MDM2*), the growth arrest, and DNA damage-inducible 45 (*GADD45*); importantly, these upregulations are dose-dependent after IR exposure [23,24,25,26] and represent blood biomarkers of IR useful for biological dosimetry purposes [27,28,29,30,31,32,33]. Although the physical dose in Gray provides accurate information of the absorbed dose, it doesn’t inform on the biological effects which are cell type-specific and also differ from one patient to another; the biological dose assessed by monitoring the induction and persistence of different radiation exposure biomarkers in human peripheral blood in vivo can inform individual patient responses [34,35,36] and potentially help with the prediction of radiation toxicity [36,37]. The detrimental effects of IR on the immune system are of great interest, and recent preclinical studies’ findings suggest that combining immunotherapy with radiotherapy could be a promising strategy for synergistic enhancement of treatment efficacy [38]. Clinical studies have demonstrated a clear association between radiation treatment and lymphopenia [39], and a biology-based optimisation of RT requires a better understanding of the response of the immune cells to the received dose. Recently, a four-dimension blood flow model was developed to estimate the dose to circulating blood cells [40], which provides better insight into the origins of radiation-induced lymphopenia. We also developed a less sophisticated dose calculation model indicating a good correlation between physically and biologically assessed doses [41].

Biologically, our current knowledge and understanding of underlying radiobiological processes is based mainly on in vitro and animal model studies. The kinetics of transcriptional response after exposure to IR have been studied in different cell types, including blood cells; however, in vivo data are sparse. A first study was performed in patients undergoing total-body irradiation where gene expression was monitored 6 h after the first 1.5-Gy fraction in patients undergoing total-body irradiation (TBI) in preparation for hematopoietic stem cell transplantation [42]. Data points in humans were rarely designed to collect samples at multiple short time points after the first fraction of RT, mainly focusing on one time-point, 2 h [43], 6 h [42], and mostly at 24 h or later during the course of radiotherapy [18,44,45] mainly for practical or ethical reasons. To address this gap in knowledge, we designed a protocol to investigate gene expression responses over time in circulating leukocytes of cancer patients treated by RT, selecting several cancer types with different radiotherapy dose distributions and different % of body exposed. We selected a panel of nine genes responsive to ionizing radiation in blood samples and we followed them after the first fraction of the treatment. We assessed the biological impact of IR on peripheral blood leukocytes, permitting a direct comparison between physical and biological dosimetry methods.

## 2. Material and Methods

### 2.1. Blood Samples for the In Vitro Time-Course

Venous blood from three healthy donors was taken at the Centre for Radiation, Chemical and Environmental Hazards, UKHSA (Chilton, UK) with informed consent and the ethical approval of the West Midlands–Solihull Research Ethics Committee (REC 14/WM/1182) for these experiments. 

From whole blood samples (20 mL per donor), human peripheral blood mononuclear cells (PBMCs) were isolated using a density gradient centrifugation of peripheral whole blood using Lymphoprep (STEMCELL Technologies, Cambridge, UK). Briefly, blood was diluted with 1:1 volume of Dulbecco’s phosphate-buffered saline (DPBS) and layered on top of the Lymphoprep solution (15 mL of Lymphoprep for 10 mL of diluted blood). After a centrifugation at 800× *g* at 22 °C for 20 min, the cloudy PBMC layer between a top layer of plasma and a bottom layer of polymorphonuclear cells was collected and transferred to a new tube to proceed with DPBS washes (300× *g* at room temperature for 10 min). After the isolation, 1.5–2 × 106 PBMCs were seeded in T25 flasks and maintained in LGM-3 culture medium (Lonza, Slough, UK) for 0–72 h at 37 °C in a humidified 5% CO2 atmosphere.

### 2.2. In Vitro Irradiation

PBMCs were exposed to 2 Gy of X-rays at room temperature (22 °C) in air on top of a Perspex platform, with a dose rate of 0.5 Gy/min, using the self-contained 250 kVp X-ray unit (CD160/1, AGO X-Ray Ltd., Aldermaston, UK) with aluminum and copper filtration (~1 mm) containing a Varian NDI-320 source (output 13 mA, 250 kV peak, 0.2 mA). Dosimetry was performed with a calibrated reference ionization chamber for the exact exposure setup used. Exposures were always monitored using a calibrated UNIDOS E electrometer and “in-beam” monitor ionisation chamber (all from PTW, Freiburg im Breisgau, Germany) located at source. Correction factors are used to calculate exact dose. Spatial dose uniformity was checked using Gafchromic EBT2 films (Vertec Scientific Ltd., Reading, UK) to ensure a homogenous dose was delivered. Once irradiated, the samples were incubated for either 1, 2, 3, 4, 5, 6, 12, 16, 18, 20, or 24 h at 37 °C with 5% CO2. Viability was measured with a Luna FL Automated Fluorescence Cell Counter (Labtech, Heathfield, UK). Viability values ranged between 0.79 and 0.80% at the time of harvest. Following incubation, PBMCs were mixed with 700 µL of QIAzol Lysis Reagent (miRNeasy kit, Qiagen Ltd., Crawley, UK) and stored at −80 °C. 

### 2.3. RNA Extraction and Reverse Transcription

miRNeasy Mini Kit (Qiagen Ltd., Crawley, UK) was used to isolate RNA from blood cells, according to the manufacturer’s instructions. The quantity of isolated RNA was subsequently determined by spectrophotometry using a ND-1000 NanoDrop (Thermo Fisher Scientific, 168 3rd Av., Waltham, MA 02451, USA) and quality was assessed using a Tapestation 2200 (Agilent Technologies, Santa Clara, CA, USA). 

### 2.4. Blood Samples from Radiotherapy Patients

The collection of blood samples from cancer patients for in vivo and ex vivo investigation study was performed at S. P. Grigoriev Institute for Medical Radiology and Oncology of the National Academy of Medical Science of Ukraine. Each patient gave written informed consent to participate to the study. Patient recruitment, including the volume of blood to be taken and the sampling scheme (i.e. blood was taken before treatment and at the following time-points after the first RT fraction, middle, and end of the RT course, Table 1) was approved by the GIMRO Bioethical Committee. The study was conducted according to the guidelines of the Declaration of Helsinki and approved by the Local Committee of Bioethics and Deontology of the State Organization S.P. Grigoriev Institute for Medical Radiology and Oncology of the National Academy of Medical Science of Ukraine (Approval Protocol No. 3 from 17 March 2020). Informed consent was obtained from all patients involved in this study. Blood samples were taken from 6 radiotherapy patients with rectal cancer (2), lung cancer (1), head and neck cancer (2), or metastases in the brain from a primary endometrial tumour (1). 

Blood samples from all patients were collected in PAXgene tubes according to the manufacturer’s protocol (Qiagen, PreAnalytiX GmbH, Hilden, Germany).

Patients were irradiated using a Linac Clinac 600c (Varian Medical Systems Inc., Palo Alto, CA, USA), 6 MeV photon beam, at the dose rate 3072 Gy/min.

Extra blood was taken prior to the beginning of the radiotherapy treatment into EDTA tubes and exposed ex vivo to 2 or 3 Gy depending on the patient’s RT dose per fraction (Table 1). Ex vivo exposures at GIMRO were performed at the same LINAC used for radiation therapy using the same dose rate as the patients. In each individual case the irradiation of EDTA tubes with blood was performed immediately before the patient’s irradiation. Some of the exposed blood was mixed with non-irradiated blood to simulate partial body irradiation (10% irradiated blood and 90% non-irradiated blood). After irradiation and mixing, the samples were kept at 37 °C for 24 h. Another blood sample was taken at 24 h after the first radiotherapy fraction into an EDTA tube and left at room temperature for 2 h to simulate the manipulation period of ex vivo samples, and transferred in PAXgene tubes (Qiagen, PreAnalytiX GmbH, Hilden, Germany). 

Within <2 h, PAXgene tubes with patients’ in vivo or ex vivo blood samples were passed to a biobank (Ukraine Association of Biobank, Kharkiv, Ukraine), where they were cooled down, frozen at −20 °C, and stored at −70 °C until shipment on dry ice to UKHSA RCE, UK, for transcriptional analyses.

### 2.5. RNA Extraction

Total RNA was extracted with the PAXgene Blood miRNA kit (Qiagen, PreAnalytiX GmbH, Hilden, Germany) using a robotic workstation Qiacube (Qiagen, Manchester, UK). The quantity of isolated RNA was determined by spectrophotometry with a ND-1000 NanoDrop and quality was assessed using a Tapestation 220 (Agilent Technologies, Santa Clara, CA, USA). 

A high-capacity cDNA reverse transcription kit (Applied Biosystems, Foster City, CA, USA) was used to prepare cDNA from 700 ng for the in vivo irradiated samples or 200 ng for the ex vivo irradiated samples of total RNA according to the manufacturer’s protocol.

### 2.6. Quantitative Real-Time Polymerase Chain Reaction

A Rotor-Gene Q (Qiagen, Hilden, Germany) with PerfeCTa MultiPlex qPCR SuperMix (Quanta Bioscience, Inc., Gaithersburg, MD, USA) was used to perform the QRT-PCR. The samples were run in triplicate in 10 μL reactions with 1 μL of the cDNA synthesis reaction, together with seven different sets of primers and fluorescent probes at 500 nM concentration each. 3′6-Carboxyfluorescein (FAM), 6-Hexachlorofluorescein (HEX), Texas red (TEX), CY5, Atto 680, and Atto 390 (Eurogentec Ltd., Fawley, Hampshire, UK) were used as fluorochrome reporters for the probes analysed in multiplexed reactions with different genes per run.

Primer sequences:

*HPRT1* F: 5′ TCAGGCAGTATAATCCAAAGATGGT 3′, R: 5′ AGTCTGGCTTATATCCAACACTTCG 3′, probe: 5′ CGCAAGCTTGCTGGTGAAAAGGACCC 3′;

*CCNG1* F: 5′ GGAGCTGCAGTCTCTGTCAAG 3′, R: 5′ TGACATCTAGACTCCTGTTCCAA 3′, probe: 5′ AACTGCTACACCAGCTGAATGCCC 3′; 

*PHPT1* F: 5′ TCGCTCTCATTCCTGATGTG 3′, R: 5′ TCGTAGATGTCCGCATGGTA 3′, probe: 5′ CTTGTAGCCGCGCACGATCTCCTT 3′;

*FDXR* F: 5′ GTACAACGGGCTTCCTGAGA 3′, R: 5′ CTCAGGTGGGGTCAGTAGGA 3′, probe: 5′ CGGGCCACGTCCAGAGCCA 3′;

*GADD45* F: 5′ CTGCGAGAACGACATCAAC 3′, R: 5′ AGCGTCGGTCTCCAAGAG 3′, probe: 5′ ATCCTGCGCGTCAGCAACCCG 3′;

*DDB2* F: 5′ GTCACTTCCAGCACCTCACA 3′, R: ACGTCGATCGTCCTCAATTC 3′, probe: 5′ AGCCTGGCATCCTCGCTACAACC 3′;

*MDM2*: 5′ CCATGATCTACAGGAACTTGGTAGTA 3′, R: ACACCTGTTCTCACTCACAGATG 3′, probe: 5′ CAATCAGCAGGAATCATCGGACTCAG 3′;

*SESN1*: 5′ GCTGTCTTGTGCATTACTTGTG 3′, R: CTGCGCAGCAGTCTACAG 3′, probe: 5′ ACATGTCCCACAACTTTGGTGCTGG 3′;

*CDKN1A*: 5′ GCAGACCAGCATGACAG 3′, R: TAGGGCTTCCTCTTGGA 3′, probe: 5′ TTTCTACCACTCCAAACGCCGGCT 3′;

*PUMA*: 5′ CGGAGACAAGAGGAGCAG 3′, R: GGAGTCCCATGATGAGATTG 3′, probe: 5′ CCCTCACCCTGGAGGGTCCTGT 3′;

During all our experiments, the reactions were performed with the same cycling conditions: 2 min at 95 °C followed by 45 cycles of 10 s at 95 °C, then 60 s at 60 °C. Data were collected and analysed using Rotor-Gene Q Series software. Hypoxanthine phosphoribosyltransferase 1 (*HPRT1*) internal control was used to normalise the gene target Ct (cycle threshold) values. For each gene, standard curves obtained from a serial dilution of PCR-amplified DNA fragments were used to convert Ct values to transcript quantity.

### 2.7. Blood Dose Calculation

The blood dose calculation used in this study was published earlier (30). Briefly, when we considered the time needed for gantry rotation, the delivery of one treatment fraction took about 2 minutes. Although the treatment planning systems are able to calculate 3D dose distribution in the human body very accurately, currently-used algorithms are not able to take blood flow into account. Thus, we used 3D dose distribution calculated by the treatment planning system Eclipse version 15.6 (Varian Medical Systems, Inc., Palo Alto, California, USA) to estimate the mean blood dose.

Our calculation method assumes that most of the blood flows through the irradiated volume during the 40–60 s-long effective irradiation period (2 min treatment period) and thus radiation dose is delivered to full blood volume.

The Eclipse software displays the geometry of the patient acquired by computed tomography and computed dose distribution. Using these data for each patient, we determined irradiated volume (*IV*) defined as a volume surrounded by 5% isodose and mean dose (*D*) in this volume. Five percent Isodose is the surface that connects points in 3D dose distribution where the dose is equal to 5% of the prescribed dose. Doses lower than 5% of the prescribed dose can be neglected. The reason for cutting off lower doses is the consistency of calculation with respect to different ranges of CT scans and treatment sites.

The patient mean blood dose (*MBD*) was calculated according to the relation (Table 2):

$$MBD=D\frac{IBV}{BBV}=D\frac{BBV\frac{IV}{V}}{BBV}=D\frac{IV}{V}$$

*MBD*—mean blood dose
*D*—irradiated volume mean dose
*IBV*—irradiated blood volume
*IV*—irradiated volume
*BBV*—body blood volume
*V*—total patient volume (approximately equal to patient weight)

We assumed that blood in the patient’s body is irradiated and stored homogeneously, and that 1 dm^3^ of human body weighs approximately 1 kg.

### 2.8. Correction Factor Calculations

In vivo sample values were corrected to 2 Gy with blood dose calculations (Table 2) in order to compare *FDXR* gene expression with ex vivo exposures. In vivo samples reflect a partial body irradiation with a dose received to the blood calculated in Table 2. Although the patients received doses per fraction of 2 and 3 Gy, circulating blood leukocytes are exposed only when present in the blood vessels localised in the irradiated volume. Patient blood samples therefore contain a mix of non-irradiated cells and cells irradiated to a range of doses accumulated during the radiotherapy session. Therefore, although the dose per fraction is 2 or 3 Gy, the dose to the blood needs to be adjusted. In the ex vivo exposures of PBMCs, the dose is homogeneous, and all the cells received a 2 Gy dose. In order to compare the cellular responses in vivo and ex vivo, we therefore adjusted the dose received, following the formula described below: In vivo corrected values= (fold change × 2 Gy)/1 fraction dose (Gy).

### 2.9. Statistical Analysis

Minitab software was used to perform statistical analyses. Data are presented as mean ± standard deviation (SD) and comparisons were analysed by *t* test (student’s *t* test). One-way ANOVA followed by Tukey’s tests was applied for multiple comparisons. A significance *p* ≤ 0.05 was applied for all tests.

## 3. Results

Six patients representing four types of cancer were selected to cover a range of volumes exposed. The detailed description of the patients’ radiotherapy treatments and blood collection time points is provided in Table 1; two laryngeal and oropharyngeal patients with irradiated area being oropharyngeal and neck, two rectal cancer patients irradiated in the pelvic area, one lung cancer patient irradiated in the upper lobe of the right lung, and one patient with a metastatic tumour subjected to whole-brain irradiation. The RT schedules represented a various number of fractions, from 10 for lung/brain (3 Gy per fraction, 30 Gy in total) to 35 oropharyngeal (2 Gy per fraction, 70 Gy).

Blood dose calculations were obtained and described in Table 2. The percentage of body part irradiated ranged from 3.5% for the brain, 3.9–4.1% for the neck area, 8.3% for the lung, and up to 16.7–19.1% for the pelvis area. This is reflected in the calculated dose per fraction to the blood of 0.038–0.045 Gy for neck, 0.077 for the brain, 0.092 for the lung, and 0.155–0.169 for the pelvis. 

**Figure 1:**
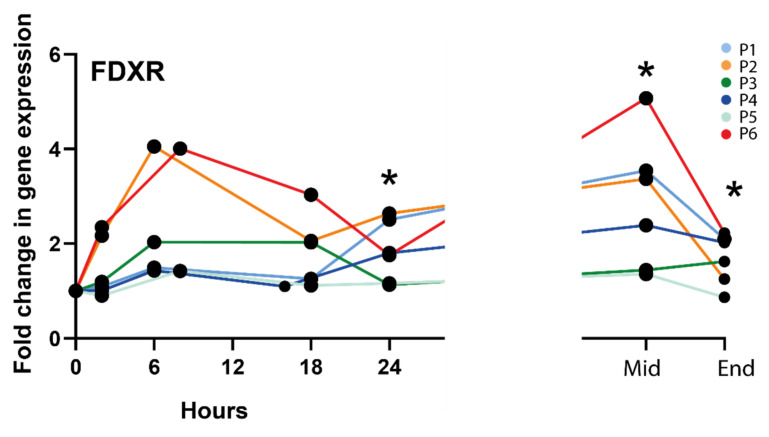
*FDXR* expression profile in all radiotherapy patients. * Significantly different from control (before treatment) (paired *t* test, *p* ≤ 0.05).

The level of expression of the genes, *FDXR, CCNG1, PHPT1, CDKN1, SESN1, PUMA, DDB2, MDM2, GADD45* was measured by quantitative PCR using RNA extracted from the RT patients’ blood samples collected at 2, 6–8, 16–18, and 24 h (Supplementary Table S1) after the first RT fraction, at the middle, and at the end of RT. Results are presented as mean +/− standard deviation across all patients. For practical reasons, one “pelvic” patient (P2) was sampled at 6 h post-RT while the second one was sampled at 8 h (P6), like for the brain tumour patient (P5); similarly, one “neck” patient was sampled at 16 h (P4) while the other one at 18 h (P1). At most early time points, except at 24 h for *FDXR*, *CCNG1,* and *CDKN1,* and 18 h for *SESN1*, the expression level was not significantly different from the control sample collected just before the beginning of RT. This is likely due to the heterogeneity of the patients and treatments, with differences in dose and volume exposed. The fold change values are significant for *CCNG1, SESN1,* and *DDB2* at the middle and end of RT for *FDXR* when the total accumulated dose to the blood is much higher, (Supplementary Table S1). Overall, *FDXR* provided the highest fold changes, reaching a maximum 2.72-fold mean at 8 h and was selected for subsequent analyses (Figure 1). 

**Figure 2:**
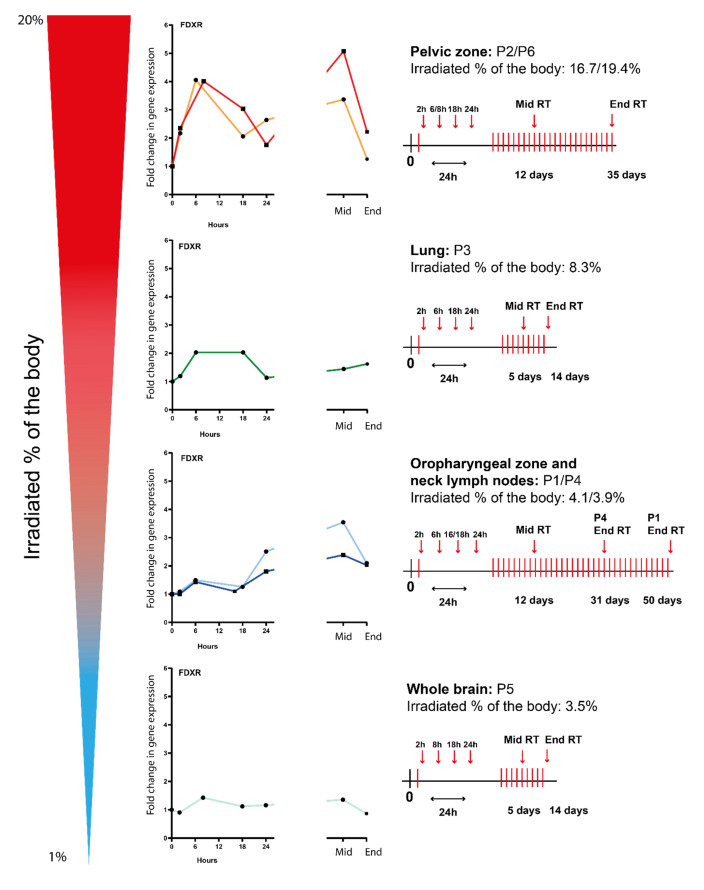
*FDXR* expression profiles of several types of radiotherapy cancer patients (i.e., lung, neck, brain, and pelvis) ranked by irradiated % of the body at 2 h, 6–8 h, 16–18 h, and 24 h after the first fraction and at the middle and end of radiotherapy. A schematic radiotherapy schedule has been included for each irradiated area.

Therefore, the *FDX*R expression profiles for all types of radiotherapy cancer patients (lung, neck, brain, and pelvis) were presented individually in decreasing order of irradiated body % (Figure 2); a schematic radiotherapy schedule has been included for each irradiated area. Following the first fraction, although the calculated physical dose to the blood was very low, an upregulation of *FDXR* gene transcription was detectable after 2 h and was clearly dependent on the dose from the lowest irradiated percentage of the body (3.5%, whole brain) to the highest, (up to 19.4%, pelvic zone) reaching a peak at 6–8 h for all patients except P1 and P4 (oropharyngeal zone and neck lymph nodes) where the maximum upregulation was at 24 h. After multiple fractions (mid-RT), the expression level increased further or reached a plateau phase and was still significantly up-regulated at the end of RT. 

**Figure 3:**
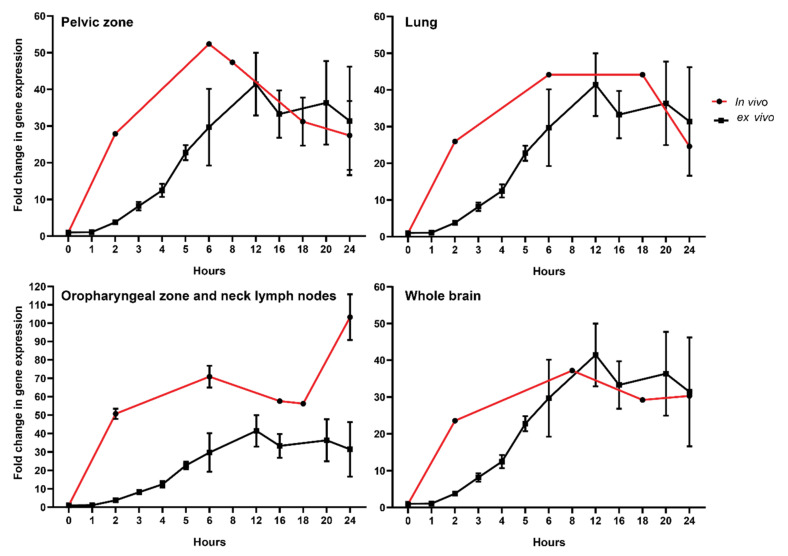
Comparison of *FDXR* responses to ionizing radiation in healthy donors’ PBMCs exposed ex vivo with RT patients’ whole blood exposed in vivo. Isolated PBMCs were exposed to 2 Gy (0.5 Gy/min) while in vivo sample values were corrected to 2 Gy using dose to the blood calculations (Table 2) as described in the material and methods section.

To compare transcriptional dynamics in vitro and in vivo, we performed experiments where PBMCs from healthy donors’ blood were incubated for either 1, 2, 3, 4, 5, 6, 12, 16, 18, 20, or 24 h after irradiation with a 2 Gy dose, used classically as the dose per fraction (Figure 3). 

To further compare directly both data sets, we corrected the in vivo data with a factor corresponding to the ratio of dose (i.e., dose to the blood calculated for in vivo samples corrected to 2 Gy used in vitro) (Figure 3). This was possible because the dose response for *FDXR* RNA expression increase linearly with the dose in this dose range both in vitro and in vivo [41]. The data were presented for each of the four RT-treated cancer types. Remarkably, the reconstructed temporal response was consistent with the one obtained in vitro. Overall, we discovered a good correlation in the kinetics of expression from the 8-hour time point onward, indicating no major differences in transcriptional changes in vitro and in vivo. The temporal peak of expression response of *FDXR* was conserved across patients. One exception was for the oropharyngeal zone and neck lymph nodes (Figure 3) where the in vivo calculated doses were associated with higher fold change in FDXR gene expression in vivo than in cells exposed in vitro. At earlier time points, however, a difference in the dynamics of RNA expression can be seen, which suggests that different mechanisms may be acting in the first hours. 

**Figure 4:**
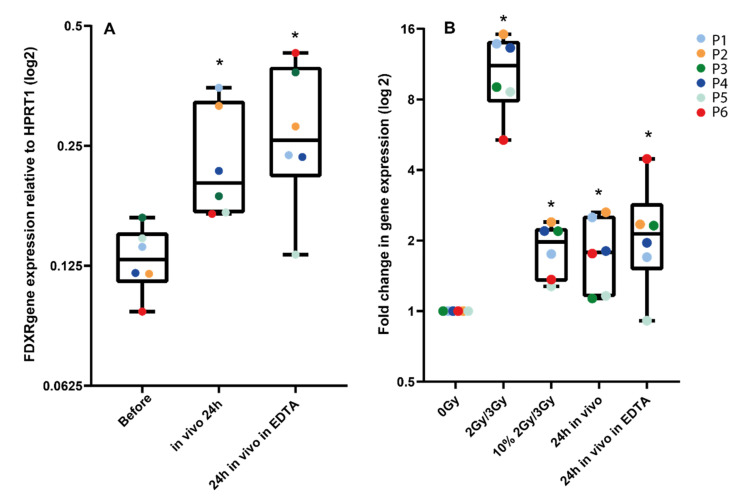
Comparison of *FDXR* transcriptional response to radiation in vivo and ex vivo from the same donor. In panel **A**, 24 h post-exposure samples taken directly into PaxGene tubes were compared to samples taken in EDTA tubes left at room temperature for 2 h, then in a PaxGene tube for storage to mimic the ex vivo sample manipulation. In panel **B**, blood collected from each patient before radiotherapy was exposed ex vivo to 2 Gy or 3 Gy, depending on the patient-specific radiotherapy schedule. These samples were compared to a diluted ex vivo exposed sample (i.e., 10% in non-irradiated blood) and to in vivo samples 24 h after exposure in PaxGene tubes or EDTA/PaxGene tubes. * Significantly different from the control (before treatment or 0 Gy) (paired *t* test, *p* ≤ 0.05).

Lastly, we compared *FDXR* transcriptional responses to radiation in vivo and ex vivo from same-donor samples (Figure 4). First, we confirmed that the fold change in transcriptional expression between blood directly drawn in a PAXGene tube and blood from the same patient taken in an EDTA tube and left at room temperature for 2 h in order to mimic ex vivo sample manipulation, and it was not significantly different (Figure 4A). Then, we compared these data to in vitro irradiated blood collected from each patient (P1–P6) before the beginning of RT and exposed ex vivo to 2 Gy or 3 Gy, depending on the radiotherapy schedule of the patient. As expected, the fold change for these high doses is much higher, reaching a mean 10.9-fold increase in expression. These samples were also compared to mixed samples [dilution of the ex vivo exposed sample (10% in 90% non-irradiated blood], mimicking the partial exposure in vivo. No significant difference was detectable, hence validating our comparison in Figure 3. 

## 4. Discussion

Circulating blood leukocytes are an attractive source of information for developing RNA expression-based biological dosimetry tests; sampling is minimally invasive and samples can be analysed using a rapid protocol of less than 4 hours [44]. Accidental radiation exposures are rarely whole-body exposures, and a partial body exposure cannot be identified in blood samples by gene expression assays where all cells are lysed together. To better understand how radiotherapy treatments affect circulating lymphocytes, Yovino et al. [45] were the first to propose a mathematical model to estimate the dose to circulating lymphocytes. More recently, Hammi and colleagues [46] have developed a computational blood flow model to calculate IR dose to the circulating blood during a course of fractionated radiotherapy [47].

At the molecular level, IR leads to DNA damage, triggering the DDR; in particular, DSBs activate the ATM/CHEK2/p53 pathway, inducing the transcription of stress genes. IR induces different DNA lesions, DSBs being the most difficult and inefficient to repair, leading to cell death, cancer, mutations, chromosomal translocations, apoptosis, and cancer [48]. During DDR activation, multiple layers of regulation and post-translational modifications of p53 modulate the induction of apoptosis and cell cycle arrest, which drive cell fate [49]. To date, the dynamic nature of this transcriptional response after the first fraction of radiotherapy has not been directly observed in vivo in humans. A clinical trial directly evaluating responses in healthy humans exposed to varying acute single doses of radiation is not ethically possible and scientists have been using non-human primates for validation in vivo of data generated in vitro. While *FDXR*, probably the most radiation-responsive gene reported thus far, is upregulated at the transcriptional level in leukocytes in vivo [41], it was unexpectedly reported to be significantly and consistently down-regulated following ionising radiation exposure in non-human primates [50]; therefore, in vivo animal models are not fully reliable for modelling effects in human.

The lack of biological data led to the development of this project. Here we reported for the first time the kinetics of transcriptional response to DNA damage in cancer patients’ circulating white blood cells during radiotherapy treatment. 

Immune cells circulate in the peripheral blood stream, and only a fraction of them are irradiated when passing through blood vessels located in the radiation field. Circulating lymphocytes only present ~2–5% of the total lymphocyte population, the majority of them being located in the spleen and lymph nodes [51]. Despite the biological complexity, we calculated the doses to the blood during the first fraction. Although our mean blood dose calculation method is relatively simple, its robustness was demonstrated in our previous study over a large range of doses [41] and patients with head and neck tumours (P1, P4 and P5) had a significantly smaller irradiated portion of the body than other patients and a less accurate mean blood dose can be assumed. Nevertheless, we validated the mean blood physical dose calculation method by comparing gene expression in in vivo and ex vivo exposed blood samples from the same patients (Figure 4). To be noted, we could not consider the level of tumour vascularization, which can potentially affect the dose to the circulating blood. Investigating how tumour vascularisation affects the dose to the circulating lymphocytes may permit a better understanding of radiation-induced lymphopenia and possibly allow for optimization of the dose, hence reducing lymphocyte depletion. The significance would depend on tumour vascularisation level and size.

The gene expression levels measured in the study represent a mean across all cells collected, and several parameters which could potentially influence the results were not considered, such as the patient blood pressure. Moreover, the role of the dose-rate would be interesting to study in the context of partial body exposure during RT; for the same dose at a lower dose-rate (e.g., during brachytherapy), irradiation time increases and radiation damage is expected to become more normally distributed amongst cells, resulting in a lower mean dose per leukocyte. A novel promising radiotherapy treatment, FLASH radiotherapy, delivers ionising radiation at ultra-high dose rates (≥40 Gy/s), reducing treatment times and radiation-induced damage to healthy tissues [52,53]. In this type of exposure, only some circulating leukocytes would be exposed, but to a much higher dose. It would be of great interest to analyse these cells in terms of *FDXR* expression in addition to viability.

It may be hypothesized that the dose received by immune cells and their response is determined by their localisation during RT. Cells in the tumour area do receive a higher dose, therefore affecting the stress gene response [19]. 

Transcriptional upregulation reported after one fraction of radiotherapy is very low. *FDXR* is the gene generating the strongest response; *FDXR* is an established radio-responsive gene in the field of biodosimetry [23,41], recognised as likely the most radiation-responsive gene in blood immune cells [54]. We therefore focused on *FDXR* responses.

*FDXR* is a mitochondrial flavoprotein which transfers electrons from NADPH to ferredoxin. p53 is a mediator of *FDXR*-dependent iron metabolism [55,56], critical for tumour suppression via iron homeostasis [57]. *FDXR* variants lead to sensorial neuropathies, damaging optic and auditory neurons [58]. Moreover, patient pathophysiological states may explain the rather unexpected differences in *FDXR* variant expression [59]. 

*FDXR* temporal transcriptional expression dynamics following IR exposure is particularly intriguing; it was previously reported in vitro in white blood cells [60] where transcript levels reached a peak 4–12 h post-irradiation (18–40-fold over controls), a similar pattern to the present in vivo data (Figure 3). The peak of expression of the time-dependent responses in vivo is intriguing and somehow unexpected. Several potential mechanisms are discussed below. Gene expression is known to vary due to cell specificity and functional differences between leukocyte types in addition to the stochastic nature of transcription [61]. The turnover of transcription pre-initiation complex is on the timescale of seconds [62] and should not play a detectable role here. 

Cell cycle affects gene expression following IR. The temporal transcriptional response has previously been assessed in stimulated T lymphocytes [63]. Exposed cells were collected between 15 min and 24 h. Many genes investigated responded rapidly to radiation, with peak expression occurring around 2–3 h post-irradiation (*CDKN1A, SESN1, ATF3, MDM2, PUMA,* and *GADD45A*). However, *DDB2*, *FDXR*, and *CCNG1* responded with slower kinetics, reaching peak expression between 5 and 24 h after exposure. Melanson et al. [64] have placed *FDXR* mRNA in a stable transcript cluster with a half-life of 4–6 h, which may explain the constant increase of the *FDXR* mRNA, as mRNA is synthesized but not degraded rapidly. Nevertheless, blood leukocytes are normally not dividing, and the cell cycle is not playing a role here.

Similar time-effect patterns were observed in cytogenetic studies involving multiple blood samplings after the RT fraction [49,65,66,67,68,69,70]. Interestingly, the pattern of radiation-induced gene expression changes in pelvic zone patients’ blood is similar to chromosome aberration yields’ kinetics in cervical cancer patients after their first RT fraction [65]. The overall recirculation time of human leukocytes in the “distributional pool” is about 12 h, [71,72,73]. However, it is unlikely that redistribution of leukocytes is a major mechanism responsible for the *FDXR* transcriptional expression fluctuations we observed both in vitro and in vivo as it does not take place in vitro. 

The kinetics of apoptotic death induced by IR in human leukocytes has been studied extensively. Apoptosis in lymphocytes is activated 12–14 h after IR either in vivo or ex vivo; it reaches a maximum at 18–24 h and reduces after 48 h; [74,75,76,77,78,79,80,81,82,83,84,85,86]. T- and B-lymphocytes are the most radiation-sensitive cells in the body [44]. Hammi and colleagues [46] reported that after the first fraction (2 Gy at 2 Gy/min), the calculated mean dose to the blood was very low, 0.004 Gy for IMRT and the volume of blood receiving any dose after one fraction was 18.4%. After 30 fractions, the highest dose to 1% of blood was calculated to be 0.343 Gy. Falcke et al. found that B-lymphocytes and natural killers (NK) were more radiosensitive than T-lymphocytes. Twenty-four hours after irradiation, these cells indicated decreases in viability by 10–15% for a dose of 0.7 Gy. Later (48 h), this decrease reached 40–50%, up to 70% at 72 h [87]. Cell death should not be contributing to the responses observed at 24 h, considering how extremely low the blood doses calculated are. It should be noted that we used isolated PBMCs for in vitro studies while all leukocytes (PBMCs plus granulocytes) were lysed in PAXGene tubes. However, no significant differences in *FDXR* expression were found between WBC and PBMCs at 24 h following a 2 Gy dose [88]. 

The patterns of *FDXR* gene expression at the middle and end of the RT course can be attributed to the features of patients’ individual RT schemes. Cytogenetic studies in vivo during the RT course reported an increase in lymphocytes yields carrying radiation markers proportional to the size of the irradiated target area and exposed body volume [89,90,91,92]. This positive dependence on the lymphatic tissues content inside the irradiated zone was also reported [93].

There is another potential cause for the shape of the time course response. There is an extensive co-regulation between splicing and transcription [94,95]; we recently reported the activation by IR of several *FDXR* transcript variants in vitro and in vivo in RT patients [59]. It is plausible that these variants have different half-lives and participate to the increase and/or decrease detected following the first IR fraction. Membrane-less nuclear speckles bodies are interchromatin granule clusters discovered as sites for splicing factor storage and modification [96]. These “splicing speckles” increase the expression of specific p53 transcription factor target genes, particularly those involved with apoptosis [97]. In the context of DNA damage, p53 selectively activates genes as part of specific programmes determining cellular outcomes [98]. It can be hypothesized that the speckle-associated p53 protein/*FDXR* gene promoter could lead to increased nascent mRNA levels following IR exposure as a response to this genotoxic stress; it could be a mechanism not yet reported, used by p53 to boost gene expression if necessary. The overall shape of the time course may be the result of the activation of several transient biological processes, (gene expression/nuclear speckles bodies activation) with intense transcription leading to a peak of expression rapidly reached (around 8 h) followed by a decrease (degradation/slower transcription rate) reaching a lower level by 24 h post-exposure. 

The ATM gene is a sensor of DNA damage [99], more specifically, IR induces DNA double-strand breaks; in turn, ATM modulates numerous signaling pathways [100]. Cultured cells from AT heterozygote carriers have been reported to have an intermediate sensitivity to radiation between normal and AT cells [101,102]. Given that 1% of the general population are AT carriers [103], a radiotherapy treatment may lead to higher leukocyte cell death, leading to immune modulation effects. The monitoring of *FDXR* expression in leukocytes during radiotherapy may therefore represent a biomarker informing on the level of ATM/CHEK2/p53 activity [63], which may affect the outcome of the treatment.

## 5. Conclusions

In this study, we demonstrated the possibility to monitor biological response kinetics of circulating immune cells to IR treatment using radiation-dependent *FDXR* RNA expression as an endpoint. The low dose of ionising radiation exposure reported leads to limited genotoxic damage and the effects of such exposures are dominated by the consequences of cell stress and damage to circulating immune cells, possibly informing inflammation-related late normal tissue toxicities. Confirmation on a larger number of patients is required to retrospectively identify patients that develop radiation toxicity (acute or late) and possibly validate this assay as a predictive biomarker of radiotoxicity. 

Monitoring inter-individual IR responses based on leukocyte RNA expression after the first RT fraction is rapid, affordable, and easy to automate [44]. Such an assay may provide clinically relevant information to develop personalised therapeutic regimens, not exclusively aiming to cure tumours, but also to adjust them to ensure an acceptable level of normal tissue adverse effects. Although the size of this study doesn’t permit us to demonstrate it, we suggest that such an assay could be adopted routinely as a tool to monitor individual biological responses. 

## Figures and Tables

**Table 1:** Description of the patients’ radiotherapy treatments and blood collection time points.

Trial ID	Cancer Type	Radiotherapy Details				Blood Collection Time Points								
						Before RT	After the 1st RTF						During RT	
		Localization of Irradiated Area	Radiation Dose per Fraction	Total Number of RTF	Total RT Dose, Gy		2 h	6 h	8 h	16 h	18 h	24 h	Mid RT (24 h after Indicated RTF Number/ RT Dose)	End RT (24 h after Indicated RTF Number/ RT Dose)
**04**	Laryngeal	Oropharyngeal zone and neck lymph nodes	2 Gy	25	50	**✓**	**✓**	**✓**		**✓**		**✓**	9 RTF/18 Gy	22 RTF/44 Gy
**01**	Oropharyngeal		2 Gy	35	70	**✓**	**✓**	**✓**			**✓**	**✓**	10 RTF/20 Gy	35 RTF/70 Gy
**06**	Rectal	Pelvic zone	2 Gy	25	50	**✓**	**✓**		**✓**		**✓**	**✓**	9 RTF/18 Gy	25 RTF/50 Gy
**02**	Rectal		2 Gy	25	50	**✓**	**✓**	**✓**			**✓**	**✓**	10 RTF/20 Gy	25 RTF/50 Gy
**03**	Lung	Right lung, upper Lobe	3 Gy	10	30	**✓**	**✓**	**✓**			**✓**	**✓**	5 RTF/15 Gy	9 RTF/27 Gy
**05**	Metastatic *	Whole brain	3 Gy	10	30	**✓**	**✓**		**✓**		**✓**	**✓**	5 RTF/15 Gy	9 RTF/27 Gy
	*—Metastases to brain from the primary endometrial tumor													

**Table 2:** Blood dose calculations.

		Total Body					More Than 5% Dose Volume				Average Blood Dose	
Trial ID	Site	Weight [Kg]	Body Blood Volume [dm^3^]	Total Dose [Gy]	Fractions	Dose Per Fraction [Gy]	Volume (=Mass) [Dm^3^ = Kg]	Irradiated Blood Volume [dm^3^]	Mean Dose [Gy] (All Fractions)	Mean Dose [Gy] (1 Fraction)	1 Fraction Dose [Gy]	Irradiated Part Of Body [%]
04	Neck	108	6.3	50	25	2.00	4.17	0.24	24.732	0.989	**0.038**	**3.9**
01	Neck	72	5.1	70	35	2.00	2.93	0.21	38.504	1.100	**0.045**	**4.1**
06	Pelvis	105	5.9	50	25	2.00	20.39	1.15	21.792	0.872	**0.169**	**19.4**
02	Pelvis	64	4.1	50	25	2.00	10.66	0.68	23.251	0.930	**0.155**	**16.7**
03	Lung	58	3.8	30	10	3.00	4.83	0.32	11.045	1.105	**0.092**	**8.3**
05	Brain	85	4.3	30	10	3.00	3.01	0.15	21.655	2.166	**0.077**	**3.5**

## Data Availability

Data available on reasonable request.

## Supplementary Materials

The following are available online at https://www.mdpi.com/article/10.3390/cancers14112649/s1, Table S1: Gene expression of *FDXR*, *CCNG1*, *PHPT1*, *CDKN1*, *SESN1*, *PUMA*, *DDB2*, *MDM2*, *GADD45* in blood of radiotherapy patients at 2, 6, 8, 16, 18, and 24 h after receiving the first radiotherapy fraction and at the middle and end of the treatment.
