# Supplementary material for: Transcriptional Dynamics of DNA Damage Responsive Genes in Circulating Leukocytes during Radiotherapy
Source: Cancers (Basel). 2022 May 26;14(11):2649. doi: 10.3390/cancers14112649 (PMC9179543; doi:10.3390/cancers14112649)
Supplement: Supplementary file 1 [file cancers-14-02649-s001.zip › cancers-1680115-supplementary.pdf]

Supplementary Materials

Table S1. Gene expression of FDXR, CCNG1, PHPT1, CDKN1, SESN1, PUMA, DDB2, MDM2, GADD45 in blood of radiotherapy patients at 2, 6, 8, 16, 18 and 24h after receiving the first radiotherapy fraction and at the middle and end of the treatment. The data are presented as mean of fold changes together with the standard deviation (SD). The presence of an asterisk (*) indicates a significant difference (paired *t*-test, $p \leq 0.05$) with the control samples (before the first RT fraction).

		**Blood Collection Time after First Fraction**							
		**2h**	**6h**	**8h**	**16h**	**18h**	**24h**	**Mid**	**End**
**FDXR**	Average	1.45	2.25	2.72	1.10	1.90	1.83 *	2.86 *	1.68 *
	SD	0.64	1.23	1.83		0.77	0.64	1.42	0.54
**CCNG1**	Average	1.05	1.03	1.47	0.91	1.11	1.14 *	1.38 *	0.98
	SD	0.17	0.20	0.14		0.24	0.11	0.30	0.22
**PHPT1**	Average	0.97	1.11	1.55	0.81	1.17	1.12	1.43	1.15
	SD	0.10	0.22	0.10		0.20	0.18	0.42	0.20
**CDKN1A**	Average	1.67	1.17	2.48	0.50	1.69	1.57*	1.98	1.64
	SD	1.27	0.57	0.95		1.27	0.35	1.64	1.51
**SESN1**	Average	1.15	1.11	1.95	0.87	1.31*	1.12	1.24*	0.98
	SD	0.19	0.34	1.03		0.11	0.25	0.22	0.26
**PUMA**	Average	1.08	0.95	1.32	0.43	1.20	1.04	1.2	1.0
	SD	0.47	0.32	0.28		0.65	0.16	0.4	0.4
**DDB2**	Average	1.02	1.34	1.42	1.08	1.13	1.20	1.47	1.28 *
	SD	0.20	0.23	0.64		0.32	0.29	0.60	0.26
**MDM2**	Average	1.09	0.86	2.37	0.73	1.05	0.97	1.01	1.02
	SD	0.27	0.07	1.64		0.31	0.16	0.20	0.20
**GADD45**	Average	1.02	0.83	1.73	0.47	1.33	1.02	1.29	1.14
	SD	0.38	0.27	0.49		0.52	0.20	0.68	0.44
